# Anti-*Proteus* Activity, Anti-Struvite Crystal, and Phytochemical Analysis of *Sida acuta* Burm. F. Ethanolic Leaf Extract

**DOI:** 10.3390/molecules27031092

**Published:** 2022-02-06

**Authors:** Nitis Smanthong, Ratree Tavichakorntrakool, Patcharaporn Tippayawat, Aroonlug Lulitanond, Porntip Pinlaor, Jureerut Daduang, Nattaya Sae-ung, Arunrat Chaveerach, Jutarop Phetcharaburanin, Patcharee Boonsiri

**Affiliations:** 1 Centre for Research and Development of Medical Diagnostic Laboratories, Faculty of Associated Medical Sciences, Khon Kaen University, Khon Kaen 40002, Thailand; s_nitis@kkumail.com (N.S.); patchatip@kku.ac.th (P.T.); arolul@kku.ac.th (A.L.); porawa@kku.ac.th (P.P.); jurpoo@kku.ac.th (J.D.); nattaya@kku.ac.th (N.S.-u.); 2 School of Medical Technology, Faculty of Associated Medical Sciences, Khon Kaen University, Khon Kaen 40002, Thailand; 3 Department of Biology, Faculty of Science, Khon Kaen University, Khon Kaen 40002, Thailand; raccha@kku.ac.th; 4 Department of Biochemistry, Faculty of Medicine, Khon Kaen University, Khon Kaen 40002, Thailand; jutarop@kku.ac.th

**Keywords:** *Proteus mirabilis*, *Sida acuta* Burm. F., anti-struvite crystal, antiurease activity, antiswarming motility

## Abstract

*Proteus mirabilis* is a significant cause of urinary tract infection that may contribute to struvite stones. Anti-infection of this bacterium and anti-struvite formation must be considered. *Sida acuta* Burm. F. (SA) has been used for the treatment of diseases related to kidneys. Therefore, we investigated the effects of the SA leaf ethanolic extract (SAEE) on growth and on virulent factors (swarming motility and urease activity) of *Proteus* *mirabilis* isolated from kidney stone formers. We also evaluated anti-struvite crystal formation and phytochemical constituents of SAEE. The minimum inhibitory concentrations (MICs) of SAEE against three clinical *P. mirabilis* isolates were 8 mg/mL. Intriguingly, the 1/2MIC of SAEE had significant inhibitory effects on the swarming motility and urease activity of clinical *P. mirabilis* isolates when compared with the condition without SAEE. The SAEE at the various concentrations significantly inhibited the average weights of struvite crystals in a dose-dependent manner, compared with the control. The phytochemical analysis revealed that SAEE contained catechin, chlorogenic acid, rutin, and ferulic acid. This study indicated that SAEE has anti-*P. mirabilis* and anti-struvite crystal activities via its bioactive compounds. For this reason, SAEE may be developed as a new agent for the treatment of struvite stone induced by *P. mirabilis*.

## 1. Introduction

Kidney stone disease (KSD) is an important urological problem worldwide, especially in the northeastern region of Thailand [1,2]. It is known that struvite (magnesium ammonium phosphate (MAP) stone is related to urinary tract infections [3]. The elevation of NH_4_^+^ concentrations, resulting from the urease activity of bacteria (e.g., *Proteus mirabilis*), causes urinary pH to increase. NH_4_^+^ binding with available cations in the urine leads to struvite crystal formation. Struvite crystals can grow rapidly in the urinary tract system. The large size of struvite stone can cause severe injury in the kidneys. Our previous report [4] showed that *P. mirabilis* isolated from kidney stone patients could induce the formation of struvite crystals in artificial urine. *P. mirabilis* is the most common urease-producing bacteria, frequently resistant to antimicrobial drugs, isolated from stone matrices of kidney stone patients [5]. *P. mirabilis* possesses numerous virulence factors that contribute to the establishment of struvite stone formation and urinary tract infections (UTIs), especially motility (i.e., flagella) and enzymes (i.e., urease) [6,7]. Flagella-mediated motility of *P. mirabilis* can ascend rapidly to the kidneys. The interpretation of urine and stone culture data should be provided for the use of antimicrobial agents in urologic stone practice [5,8].

A possibility for the treatment of struvite stone is the use of antibacterial and anticrystal agents to prevent the deposition of struvite crystals into the kidney collecting system. Plants are important and available resources for primary health care in conventional medicines, including Thai folk medicine. *Sida acuta* Burm. F. (SA) has been used for the treatment of several diseases such as kidney stones and urinary disease treatment (diuretic) [9,10]. Our previous study reported that SA leaf aqueous extract (SAAE) contained phenolic compounds (i.e., *p*-hydroxybenzoic acid, ferulic acid, and resveratrol) and showed antibacterial activity on several bacterial reference strains [11]. SAAE may have the potential to be used as an alternative medicine for the prevention and treatment of KSD with UTIs. As the SAAE could not inhibit *P. mirabilis* DMST 8212 at 32.00 mg/mL, we developed the SA leaf ethanolic extract (SAEE). The effect of SAEE on *P. mirabilis* isolated from kidney stone matrices and their virulent factors related to struvite formation needs to be verified.

The aims of this study were to (1) investigate the inhibitory effects of SAEE on growth, swarming motility, and urease activity of *P. mirabilis* isolated from kidney stone formers, (2) evaluate the inhibitory effect of SAEE on struvite crystal formation, and (3) investigate phytochemical constituents of SAEE.

## 2. Results

### 2.1. Minimum Inhibitory Concentration (MIC) of SAEE on P. mirabilis

The MIC of gentamicin against *Escherichia* *coli* ATCC 25,922 (a reference strain) was in the quality control range of CLSI. The MICs of SAEE against all three clinical isolates of *P. mirabilis* were 8.00 mg/mL, while *P. mirabilis* ATCC 25933 was 16.00 mg/mL The MICs of gentamicin ranged from <0.12 to 4.00 µg/mL.

### 2.2. Inhibitory Effect of SAEE on Swarming Migration of P. mirabilis

**Figure 1:**
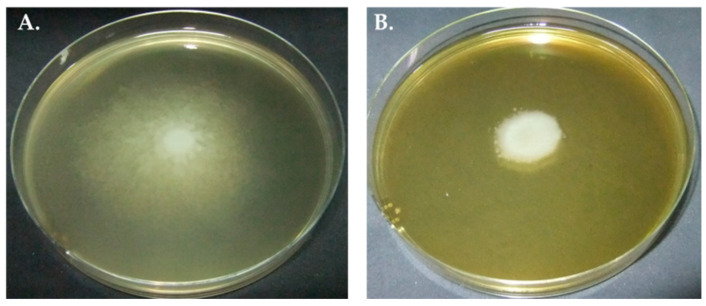
Effect of SAEE on swarming motility of *P. mirabilis* in conditions without (**A**) and with (**B**) 1/2MIC of SAEE.

At 1/2MIC of SAEE, there were significant inhibitory effects on the swarming motility of all *P. mirabilis* isolates when compared with the condition without SAEE (P-value < 0.05) (Figure 1 and Table 1). The percent reductions in the migration distance (cm) of the swarming motility of *P. mirabilis* strains PMKS1, PMKS2 and PMKS3 were 26.86, 20.71, and 71.60, respectively, while *P. mirabilis* ATCC 25933 was 80.61.

### 2.3. Inhibitory Effect of SAEE on Urease Activity of P. mirabilis

At MIC of SAEE, there were significant inhibitory effects on urease activity of PMKS1 and PMKS2 when compared with the conditions without SAEE (*p*-value < 0.05) (Table 2). The percent inhibitions in the urease activity of *P. mirabilis* strains PMKS1, PMKS2 and PMKS3 were 35.75, 39.48, and 15.29, respectively, while *P. mirabilis* ATCC 25,933 was 47.91.

### 2.4. Inhibition of Struvite Crystal Weights by SAEE

On day 15, the morphologies of struvite crystals in the hydrogel medium were formed in different crystal shapes such as dendrite, needle, X-like, and rectangle. The weights of struvite crystals were expressed as median (IQR1, IQR3) (Table 3). There was a statistically significant difference between groups, as determined by the Kruskal–Wallis ANOVA (chi-squared = 15.25, *p*-value = 0.0011). A Tukey post hoc test was statistically significant in the effectiveness of SAEE.

### 2.5. Identification and Confirmation of Struvite Crystal

**Figure 2:**
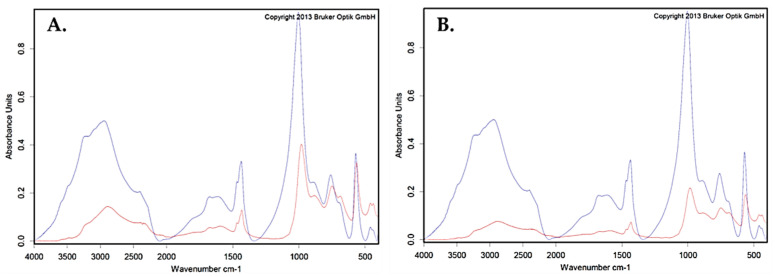
Search for FTIR spectrum result of two crystal samples in conditions without (**A**) and with (**B**) 32 mg/mL of SAEE from Bruker kidney stone database. The main component of the crystal was clearly identified as struvite. The blue and red lines represent the reference and sample spectra, respectively.

The chemical compositions of all crystal samples at day 15 were analyzed in triplicate in each SAEE concentration (0–32 mg/mL) by Tensor-II Attenuated Total Reflection Fourier-transform infrared spectroscopy (ATR-FTIR) spectroscopy (Figure 2). The analysis of all crystal samples (red curve) with struvite as the main component (95%) had an excellent hit quality point (blue curve). Additionally, the substance “carbonate apatite” was detected as a second component (5%).

### 2.6. Determination of Phytochemical Constituents of SAEE

**Figure 3:**
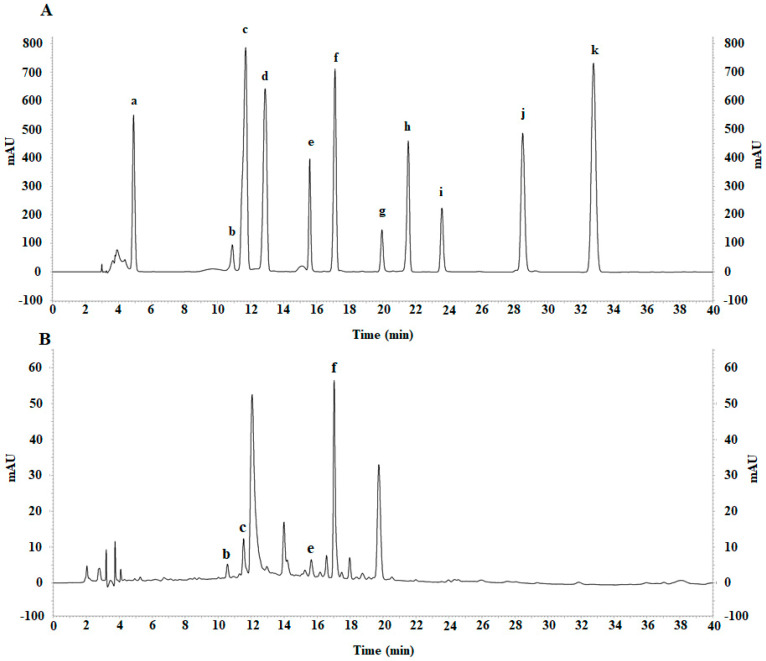
HPLC chromatograms of standard phytochemicals (**A**) and SAEE (**B**). a: gallic acid, b: catechin, c: chlorogenic acid, d: caffeic acid, e: rutin, f: ferulic acid, g: myricetin, h: resveratrol, i: quercetin, j: kaempferol and k: eugenol.

The yield of the SAEE was 17.48%. The HPLC retention times (min) of standard phytochemicals were 4.91 (gallic acid), 10.89 (catechin), 11.60 (chlorogenic acid), 13.04 (caffeic acid), 15.61 (rutin), 17.19 (ferulic acid), 19.97 (myricetin), 21.69 (resveratrol), 23.70 (quercetin), 28.70 (kaempferol), and 32.70 (eugenol). The SAEE showed peaks that represent catechin, chlorogenic acid, rutin, and ferulic acid at concentrations of 572.61 ± 6.46, 480.48 ± 32.77, 492.62 ± 40.94, and 1026.95 ± 9.58 µg/g dry weight, respectively (Figure 3).

## 3. Discussion

*P. mirabilis* is a cause of serious UTIs and struvite stones that are associated with its swarming motility and urease activity [3,4,12]. Urease is an important virulence factor for infection-induced stone formation. The enzyme hydrolyzes urea to carbon dioxide and ammonia. Ammonia results in an elevation of the pH of human urine, and normally soluble polyvalent anions and cations precipitate at high pH to form struvite and carbonate apatite crystals in the urinary tract [3]. The dual actions of some medicinal agents against *P. mirabilis* and struvite crystals need to be elucidated.

Natural plants have gained considerable importance in medicine around the world, including in Thailand. SA is a weed found in subtropical and tropical areas. It has been used in Indian traditional medicine for several diseases such as kidney stone and urinary diseases treatment (diuretic) [9,10]. Moreover, the other previous report [13] of acute toxicity test showed that there was no death recorded in mice dosed with SAEE at 5000 mg/kg body weight. No significant changes were observed in body weight and behaviour within 24 h. Our previous study reported that SAAE contained the phenolic compounds (i.e., p-hydroxybenzoic acid, ferulic acid, and resveratrol) and showed antibacterial activity on several bacterial reference strains [11]. Therefore, we used three clinical strains of *P*. *mirabilis* isolated from stone matrices in this study. The results showed that the MICs of SAEE could inhibit all three *P. mirabilis* isolates at 8 mg/mL, whereas a previous study showed that MIC of the SAAE could not inhibit *P. mirabilis* DMST 8212 at 32.00 mg/mL [11]. This difference may be due to different factors—namely, (1) solubility of bioactive agents in the extracting solvents, (2) the number of bioactive agents in SA depending on harvest season, and (3) strain of *P. mirabilis* (the antimicrobial susceptibility test of theses isolates was performed by disc diffusion method. All of them were susceptible to nine antimicrobial drugs—amikacin, ampicillin, cephalothin, sulfamethoxazole/trimethoprim, gentamicin, norfloxacin, ofloxacin, cefotaxime, and ceftazidime—except PMKS2, which resisted ampicillin and sulfamethoxazole/trimethoprim [5]). Our finding is consistent with another study indicating that SAEE had a significantly higher antimicrobial activity than SAAE due to the solubility of bioactive agents in the extracting solvents [14]. In our study, SAEE had significant inhibitory effects on swarming motility and on the urease activity of *P. mirabilis* isolated from kidney stone formers. Notably, variations in the percentages of inhibition for swarming motility and urease activity could be due to the variation of *P. mirabilis* strain in clinical isolates. 

Some phenolic compounds may relate to the inhibition of nucleic acid synthesis, energy metabolism, cell attachment, attenuation of the pathogenicity, and membrane functions [15,16]. From the phytochemical analysis, SAEE contained phenolic compounds including catechin, chlorogenic acid, ferulic acid, and rutin. In vitro experiments demonstrated the antibacterial effects of catechin, chlorogenic acid, and ferulic acid on both Gram-positive and Gram-negative bacteria [17]. An inhibitory molecular mechanism of catechins against some bacteria may be involved with plasma cell membrane damage by intercalating the lipid bilayer [18]. Chlorogenic acid may exert the physiological change at bacterial membrane leading to cell death [19]. Ferulic acid may act on the surface properties of the bacterial cell membrane by developing local rupture or pore formation, resulting in leakage of essential intracellular constituents [20]. In addition, rutin was reported to have an effective antibacterial activity against *P*. *vulgaris* [21]. The proposed mechanism of rutin is to prevent folic acid synthesis [22] and rutin acts as a urease inhibitor [23]. The present study indicated that SAEE could affect the growth and virulent factors (swarming motility and urease activity) of *P. mirabilis*. This study was performed in vitro and 32 mg/mL of SAEE showed modest potency for the anti-struvite crystal. The phenolic compounds of SAEE may play significant inhibitory roles by a synergistic effect of multiple components in the crude extract of SA. The proposed mechanism of SAEE on anti-struvite crystallization may be due to the interaction between the bioactive compounds and struvite crystals or urease, resulting in a reduction in crystal growth. 

The SAEE could inhibit the growth, swarming motility, and urease activity of *P. mirabilis*, and the growth of struvite crystal via its bioactive compounds. Therefore, it is interesting to deeply investigate this weed as pharmaceutical raw material. However, the inhibitory effects of SAEE on the growth of *P. mirabilis* and struvite crystal formation should be further analyzed in vivo. 

The limitation in this study is that only three isolates of *P. mirabilis*—PMKS1, PMKS2, and PMKS3—were tested for antibacterial, antiswarming motility, and antiurease activity. These isolates were obtained from 100 stone matrices; of those, five isolates of *P. mirabilis* were found, and three of them were satisfied for this experiment. This study was also limited by a few numbers of standard phytochemical compounds, which were injected in HPLC, compared with SAEE. Therefore, the three major peaks that appeared in the HPLC chromatogram were unidentified. However, the peak at retention time 12 min, eluted from a C18 reverse phase HPLC column, may be due to a compound with higher polarity, compared with peaks at retention times 14 min and 19 min. Moreover, they might have functional groups with polarity, such as the OH group, because they came out after chlorogenic acid. All of these three peaks might act as bioactive compounds. This should be investigated in further study.

## 4. Materials and Methods

### 4.1. Ethics

This study was conducted following the Declaration of Helsinki and approved by the Institutional Ethical Committee of Khon Kaen University, Khon Kaen, Thailand (Approval Nos. HE 521177and HE 581501). Written informed consent was obtained from all participants for their clinical specimens.

### 4.2. Sample Collection and Selection Criteria

Stone samples were collected from a total of 100 kidney stone formers and the bacteria cultured. Those with underlying systemic diseases and secondary causes of nephrolithiasis, e.g., primary hyperparathyroidism, renal tubular acidosis, active UTIs or other infections within 1 year prior to admission, and history of antibiotic treatment of UTIs within 1 year prior to admission were excluded [5]. Among them, five subjects had positive *P. mirabilis* cultures from their stone samples. However, only 3 isolates of *P. mirabilis* (PMKS1, PMKS2, and PMKS3) were satisfactory for further study.

### 4.3. Determination of the Antibacterial Activity of SAEE

Antibacterial activity of SAEE, at the concentration range 0–32 mg/mL, was studied. Minimum inhibitory concentrations (MICs) of this extract for three *P. mirabilis* isolates from stone matrices were also determined by the broth microdilution method [24]. *E*. *coli* ATCC 25922, and *P. mirabilis* ATCC 25933 (reference strains) and gentamicin against the reference strains were used as controls. 

### 4.4. Inhibitory Effect of SAEE on Swarming Motility of P. mirabilis

Luria–Bertani broth (Oxoid, England) with 1.5% agar was used as the test medium in conditions without and with 1/2MIC of SAEE. Each bacterial culture was adjusted to 0.5 McFarland. Five microliters of each bacterial suspension were dropped onto the center of a swarming agar plate and incubated at 37 °C for 24 h. The swarming motility was measured as migration distance (cm) [25]. *P. mirabilis* ATCC 25933 (a reference strain, and swarming motility bacterium) was used as a positive control.

### 4.5. Inhibitory Effect of SAEE on Urease Activity of P. mirabilis

Each *P. mirabilis* isolate was cultured overnight in conditions without and with 1/2MIC of SAEE. The bacterial cells were centrifuged (10 min, 10,000 g), washed with phosphate-buffered saline, and sonicated at 30% amplitude with 30 s intervals on ice by using a sonicator (model Q55, Qsonica, Newtown, CT, USA). The cell lysate supernatants had their urease activity determined by an assay kit (MAK120, Sigma-Aldrich, Saint Louis, MO, USA) and measured at 670 nm. This enzymatic assay was calculated and expressed as unit/L. *P*. *mirabilis* ATCC 25933 (a reference strain, and urease-producing bacterium) was used as a positive control.

### 4.6. Inhibition of Struvite Crystal Formation by SAEE

Sodium metasilicate (Sigma-Aldrich, USA) solution with a specific gravity of 1.05 was mixed with 0.5 M ammonium dihydrogen phosphate (Sigma-Aldrich, MO, USA) in tubes to set the gel formation. After gelation was obtained, 20 mL of the supernatant solution of pure 1 M magnesium acetate tetrahydrate (Sigma-Aldrich, MO, USA) was mixed with SAEE to the final concentration (0–32 mg/mL) of the SAEE, and gently poured on top of the gel. Gel without SAEE was used as a negative control. After the completion of the experiment (15 days), the crystals were removed and weighed [26].

### 4.7. Identification of Struvite Crystals

Crystal samples from each concentration were collected and their chemical compositions were investigated by Tensor-II Attenuated Total Reflection Fourier-transform infrared spectroscopy (ATR–FTIR) spectroscopy (Bruker Optic, Ettingen, Germany). The powdered crystals (9.4 mg) were applied on a diamond crystal attenuated total reflectance accessory. Then, infrared spectra were recorded in the wavelength range from 4000 to 400 cm^−1^ at 4 cm^−1^ resolutions, accumulating 32 scans per spectra. Triplicates of each sample were analyzed by OPUS program version 7.2 for Windows (Bruker Optic GmBH, Germany) and calculated compared to the Bruker kidney stone database.

### 4.8. Extraction of SA Leaves

The SA leaves were collected from Khon Kaen Province, Thailand, and identified by Prof. Dr. Arunrat Chaveerach. After washing and drying the leaves at 50 °C, they were ground into a fine powder and extracted with 95% ethanol (Bangkok alcohol, BKK, Thailand) on a stirrer for 24 h. The SA filtrate was collected after passing through filter paper no. 1 (Whatman, Kent, UK), followed by evaporating at 40–50 °C (Rotavapor R-3; Oldham, UK) and drying at 50 °C. The crude extract of SA was stored in a desiccator with light protection for further analysis.

### 4.9. Analysis of SAEE Constituents Using High-Performance Liquid Chromatography (HPLC)

The HPLC experiment was performed according to a previous study with some modifications [27]. The standard phytochemical compounds, including gallic acid, catechin, chlorogenic acid, caffeic acid, rutin, ferulic acid, myricetin, resveratrol, quercetin, kaempferol, and eugenol (Sigma-Aldrich, MO, USA) were used as standard compounds. The SAEE and standard compounds were dissolved in methanol (Merck, Darmstadt, Germany) at a concentration of 1 mg/mL each. Then, 100 μL of each sample was passed through a 0.20 μm particle size filter before injection into a C18 reversed-phase column (GL Sciences, Tokyo, Japan) using auto-injection (SIL-20A, Shimadzu, Tokyo, Japan). Acetonitrile (A) (Merck, Darmstadt, Germany) and 1% acetic acid (B) (RCI Labscan, BKK, Thailand) were used as the mobile phase at a flow rate of 1 mL/min using an LC-20A pump (Shimadzu, Tokyo, Japan). The mobile phase was run using gradient elution: 65% solvent B (0–15 min); 60% solvent B (15–28 min); 40% solvent B (28–40 min). The eluted compounds were detected using a UV detector (SPD-M20A, Shimadzu, Tokyo, Japan) at 280 nm. Peak areas of these compounds were compared with the standard compounds. The phytochemicals in the SAEE were calculated as µg/g dry weight.

### 4.10. Statistical Analysis

Each experiment was performed in triplicate. The normality tests were assessed by the Shapiro–Wilk test. Student’s *t*-test and Wilcoxon rank-sum test were used to assess normally and nonnormally distributed continuous data outcomes, respectively. The normal distribution data were expressed as mean ± standard deviation (SD), while the nonnormal distribution data were expressed as median with interquartile range (IQR). Kruskal–Wallis ANOVA and pairwise comparisons for the Tukey post hoc test were used to analyze which pairs of groups differ significantly. *p*-value < 0.05 was considered significant. All statistical analyses were performed using STATA version 10.1 software (STATA Corp., College station, TX, USA).

## 5. Conclusions

This study indicated that SAEE has both anti-*P. mirabilis* and anti-struvite crystal at the same concentration. It also shows antivirulent factors including swarming motility and urease activities. The bioactive compounds of SAEE may be developed as a new agent to treat infection by *P. mirabilis* or to prevent the struvite crystal formation induced by *P. mirabilis*.

## Figures and Tables

**Table 1:** Effect of SAEE on swarming motility of *P. mirabilis*.

*P*. *mirabilis* Strains	Distance of Swarming Motility in Conditions (cm), Median (IQR1, IQR3)		Inhibitory Effect (%)
	Without SAEE	With 1/2MIC of SAEE	
PMKS1	8.45 (8.40–8.50)	6.18 (5.84–7.51)	26.86 *
PMKS2	8.45 (8.35–8.55)	6.70 (4.79–6.82)	20.71 *
PMKS3	8.52 (8.47–8.58)	2.42 (2.01–3.10)	71.60 *

Abbreviation; SAEE: *Sida acuta* Burm. F. leaf ethanolic extract * = statistically significance (*p*-value < 0.05 when compared with without SAEE).

**Table 2:** Effect of SAEE on urease activity of *P. mirabilis*.

*P. mirabilis* Strains	Urease Activity in Conditions (unit/L), Median (IQR1, IQR3)		Inhibitory Effect (%)
	Without SAEE	With 1/2MIC of SAEE	
PMKS1	9.37 (8.86–9.49)	6.02 (5.22–7.29)	35.75 *
PMKS2	13.12 (11.63–13.43)	7.94 (7.22–8.76)	39.48 *
PMKS3	8.24 (6.25–8.69)	6.98 (5.98–7.71)	15.29

Abbreviation; SAEE: *Sida acuta* Burm. F. leaf ethanolic extract * = statistically significance (*p*-value < 0.05 when compared to without SAEE).

**Table 3:** The weights of struvite crystals in different SAEE concentrations (0–32 mg/mL).

Concentration of SAEE (mg/mL)	Weights of Struvite Crystals (g), Median (IQR1, IQR3)	Significance
0	1.0470 (1.0437, 1.0668)	
1	1.0392 (1.0367, 1.0457)	*
2	1.0340 (1.0203, 1.0404)	*
4	1.0363 (1.0224, 1.0390)	*
8	1.0292 (1.0262, 1.0348)	*
16	1.0255 (1.0232, 1.0344)	*, **
32	1.0137 (1.0090, 1.0155)	*, **, ^#^, ^&^

Abbreviation; SAEE: *Sida acuta* Burm. F. leaf ethanolic extract * = *p*-value less than 0.05 compared with control (without SAEE). ** = *p*-value less than 0.05, compared with SAEE (1 mg/mL). ^#^ = *p*-value less than 0.05 compared with SAEE (2 mg/mL). ^&^ = *p*-value less than 0.05 compared with SAEE (4 mg/mL).

## Data Availability

All data are available in this publication.

## Sample Availability

Samples of the compounds are available from the authors.
